# Balanced Chromosomal Rearrangement in Recurrent Spontaneous Abortions: A Case Report

**Published:** 2012

**Authors:** Ahmadreza Zarifian, Zeinab Farhoodi, Roya Amel, Salmeh Mirzaee, Mohammad Hassanzadeh-Nazarabadi

**Affiliations:** 1 *Student Research Assembly, Mashhad University of Medical Sciences, Iran.*; 2 *Department of Medical Genetics, Faculty of Medicine, Mashhad University of Medical Sciences, Iran.*

**Keywords:** Chromosomal abnormality, spontaneous abortion, chromosomal translocation, recurrent miscarriage, case report

## Abstract

One of the major causes of spontaneous abortion before the fourth month of pregnancy is chromosomal abnormalities. We report an unusual case of a familial balanced chromosomal translocation in a consanguineous couple who experienced 4 spontaneous abortions. Chromosomal studies were performed on the basis of G-banding technique at high resolution and revealed 46, XX, t (16; 6) (p12; q26) and 46, XY, t (16; 6) (p12; q26) in both partners, which induced such pregnancy complications.

Chromosomal balanced translocation is one of the most common causes of recurrent spontaneous abortions (RSA). In such cases prenatal diagnosis (PND) during the 16^th^ week of gestation is strongly recommended.

Miscarriage, also known as spontaneous abortion, is the most common complication of pregnancy. Spontaneous abortion is defined as a pregnancy which ends spontaneously before the fetus has reached viable gestational age. Therefore, the term includes all pregnancy losses from the conception until the 24^th^ week of gestation (1). Recurrent spontaneous abortion (RSA), which occurs in approximately 1-5% of conceptions, is defined as having three or more consecutive spontaneous miscarriages in the first trimester of gestation (2, 3). 

Numerous risk factors are associated with an increased risk of pregnancy loss, including: genetic and endocrine abnormalities, immune dysfunction, advanced maternal age, previous spontaneous abortions, past obstetrical history, gravidity, short interpregnancy interval in multigravidas, prolonged ovulation to implantation period and balanced chromosomal rearrangements (4-7).

Fetal aneuploidy is the reason for the majority of miscarriages before 10^th^ week of gestation. Most human aneuploidies have their roots in errors during the ﬁrst meiotic division of the oocyte, which is initiated before the time of birth and is incomplete until ovulation (8).

Over 50% of all miscarriages occur due to chromosomal abnormalities among which, trisomy is the most frequent. The most probable reason for these abnormalities seems to be advanced maternal age, which nowadays affects most of conceptions. This alteration in the reproductive behavior of couples, indubitably affects genetic abnormalities of the fetus and may increase the rate of trisomy in these pregnancies (4, 7). By contrast, few fetal trisomies have been shown to stem from paternal meiotic errors during spermatogenesis (8, 9). 

In approximately 4% of couples with recurrent miscarriages at least one partner is a carrier for either a balanced reciprocal translocation or a Robertsonian translocation. Carriers of balanced translocation are phenotypically normal but their gametes are genetically unbalanced due to meiotic errors (10).

In this study a couple with a history of recurrent spontaneous abortion was assessed to search for balanced chromosomal rearrangement.

## Case Report

**Fig 1:**
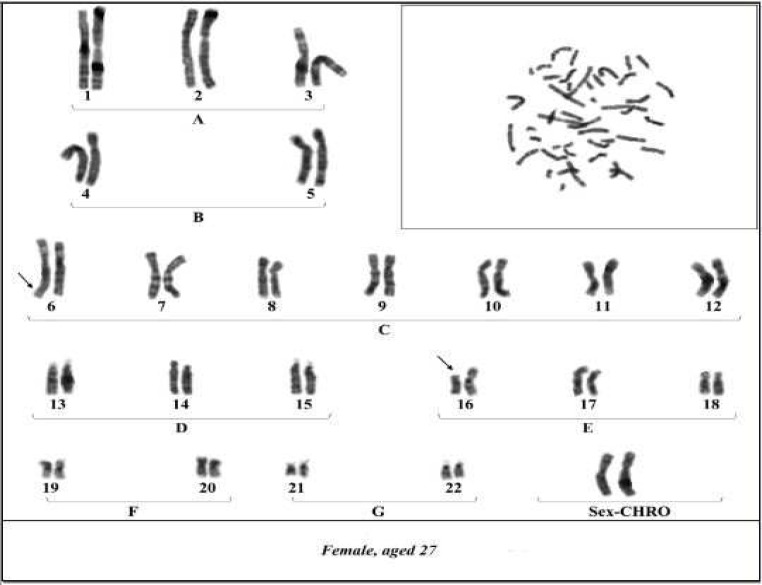
Karyotype of female partner

**Fig 2:**
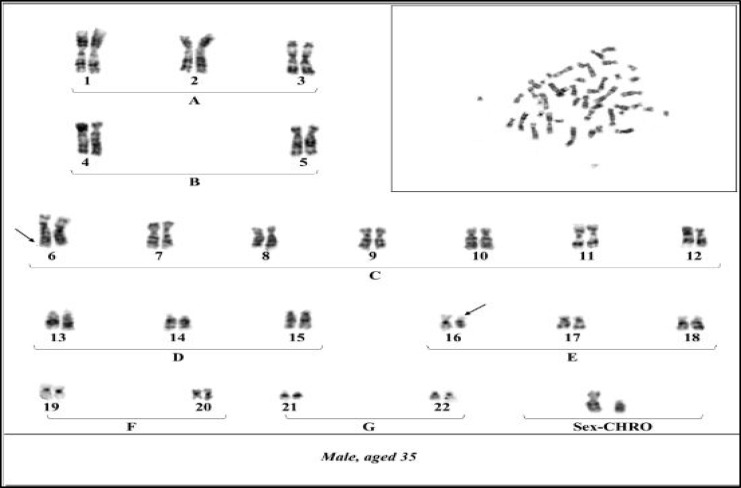
Karyotype of male partner

In this case we report a history of recurrent spontaneous abortions of a couple with unknown cause. The 35-year-old man and 27-year-old woman who had a consanguineous marriage had four intrauterine fetal deaths (IUFD) before the 16^th^ week of pregnancy. They were initially referred to the infertility center for investigation. Hormonal and anatomical factors of the uterus were normal and the mother had no underlying disease related to such abortions, therefore, they were referred to medical cytogenetic laboratory for chromosomal analysis. Chromosomal studies were performed on the basis of G-banding technique at high resolution. The results showed similar balanced chromosomal translocations between the short arm of chromosome 16 and the long arm of chromosome 6 for both partners [46, XY, t (16; 6) (p12; q26) and 46, XX, t (16; 6) (p12; q26)] with normal phenotypes (Fig 1, 2).

## Discussion

Couples who carry balanced chromosomal rearrangements can produce abnormal gametes with unbalanced chromosomal rearrangement during gametogenesis and transfer this abnormality to their fetus, which may result in either RSA or congenital abnormalities (11-13).

The frequency of uterine abnormalities in women with RSA varies from 2% to 37% and this may play an important role in failure of pregnancy. Therefore, surgical correction of uterine by either open surgery or hysteroscopic methods would contribute to more successful pregnancies (14).

In theory, only 25% of gametes of each partner are normal, therefore, prenatal diagnosis (PND) for searching karyotype of the fetus, preimplantation genetic diagnosis (PGD) and embryo donation would be suggested. Nevertheless, each of the mentioned methods would have various consequences (15, 16).

A previous study showed that the most frequent problem in balanced translocation carriers is increased frequency of miscarriage as a result of the formation of unbalanced gametes. The study also emphasized that carriers of balanced translocation must be followed for life and directed to preimplantation genetic centers to avoid fetal abnormalities (17).

Due to the hereditary transmission of this chromosomal abnormality, cytogenetic analysis for all their siblings and genetic consultation before marriage is highly recommended (18).
